# Chitinase-3-Like Protein 1 (YKL-40) Reflects the Severity of Symptoms in Atopic Dermatitis

**DOI:** 10.1155/2017/5746031

**Published:** 2017-06-04

**Authors:** Joanna Salomon, Łukasz Matusiak, Danuta Nowicka-Suszko, Jacek C Szepietowski

**Affiliations:** Department of Dermatology, Venereology and Allergology, Wrocław Medical University, Wrocław, Poland

## Abstract

Chitinase-3-like protein 1 (YKL-40) is suggested to be associated with type 2 T helper response and atopy. The aim of the study was the evaluation of serum YKL-40 level in atopic dermatitis. The study was performed on 59 patients: 27 males and 32 females, aged from 18 to 64 years. The severity of the disease was assessed by the SCORAD and objective SCORAD indexes. The severity of pruritus was measured by the visual analogue scale. Blood samples were taken to examine serum level of YKL-40, total IgE level, C-reactive protein level, white blood cell count, and neutrophil count. YKL-40 serum levels were significantly higher in patients with atopic dermatitis compared to the controls. There was a positive correlation between YKL-40 concentration and SCORAD, objective SCORAD, and pruritus. This study has shown that YKL-40 serum level is increased in patients with atopic dermatitis and reflects the severity of symptoms.

## 1. Introduction

Atopic dermatitis (AD) is an inflammatory skin disease of multifactorial and complex pathogenesis associated with a higher risk of IgE-dependent sensitization. The development of symptoms depends on the genetically conditioned impairment of the epidermal barrier, the coexistence of immune deviations, and the influence of environmental factors [1]. Many dysregulations within both the innate and adaptive immune systems have been observed in AD patients, with a special emphasis placed on the role of type 2 T helper lymphocytes. A typical feature of AD is the presence of T cells in the lesional skin. These may be type 2 innate lymphoid cells, accumulated as a result of toll-like receptors activation, as well as specifically sensitized Th2 cells in patients with allergic inflammation after allergen challenge [2–4]. These cells are the main source of type 2 cytokines, such as interleukins IL-5 and IL-13, critical in allergic response [2]. Some clinical studies have proven that specific immunotherapy targets Th2 cells, which also indicates their contribution to the pathogenesis of AD [5, 6].

Despite the growing understanding of the pathomechanisms of AD, a specific parameter that could easily be measured has not yet been found. Chitinase-3-like protein 1 (YKL-40) is one of the 18 glycosyl hydrolases, the conservative family of chitinases in mammals [7]. However, due to several mutations, YKL-40 lacks the chitinolytic enzyme activity and its biological role is unknown. It is expressed and released by many inflammatory cells, such as macrophages, neutrophils, chondrocytes, fibroblasts, endothelial cells, and smooth muscle cells [8, 9]. That is why it is suggested that this protein plays a role in inflammation, tissue remodeling, angiogenesis, and proliferation [8–12]. Moreover, some reports demonstrate that YKL-40 is associated with atopy and contributes to both innate and adaptive type 2 immune mechanisms [13–16]. This protein has been already evaluated in atopic asthma and rhinitis, demonstrating its role in these diseases [17–19]. That is why we have decided to explore that issue in skin atopy. To the best of our knowledge, there are no reports concerning the issue of YKL-40 in AD. Therefore, this is the first study further examining this matter.

## 2. Materials and Methods

This prospective study was performed on a group of 59 patients with atopic dermatitis, including 27 males and 32 female patients, aged from 18 to 64 years (mean 32.8 ± 11.2 years). In all patients, diagnosis was confirmed on the basis of clinical criteria established by Hanifin and Rajka [20]. The disease duration ranged from 0.5 to 59 years (mean 18.7 ± 13.5 years). Over a half of the patients (*n* = 33, 55.9%) suffered from other atopic disorders, such as asthma (*n* = 6) and rhinitis (*n* = 20) or both (*n* = 7). The severity of the disease was assessed using the SCORAD index [21]. The values of the SCORAD varied from 12.8 to 85 points (mean 49.1 ± 16.3 points). We also calculated the objective SCORAD, which reflects only the severity of skin changes, excluding the self-assessment of pruritus and sleep loss. The objective SCORAD rates ranged from 8.8 to 76 points (mean 40.9 ± 14.5 points). For the evaluation of the severity of pruritus, the VAS (visual analogue scale) was used, in which the possible results could range from 0 to 10 points in patients' self-assessment. The pruritus, present in the preceding 24 hours, was graded between 0.8 and 10 points (mean 6.7 ± 2.2 points). The clinical characteristics of the examined group are summarized in Table 1. All patients had a negative history of any significant comorbidities or treatments that could have had an impact on the study results. The control group consisted of 37 healthy, nonatopic volunteers—blood donors and staff members of our department—with a negative history of atopic diseases, matched for gender and age.

To evaluate the serum concentration of YKL-40, blood samples were taken from all the patients. Furthermore, some other biomarkers were investigated, such as total IgE level, CRP (C-reactive protein), ESR (erythrocyte sedimentation rate), white blood cell count (WBC), and neutrophil count. For serum YKL-40 measurements, samples of venous blood were collected, then the serum was separated and kept frozen at the temperature of −70°C until analysis. The evaluations were performed with enzyme-linked immunosorbent assay (ELISA) by R&D systems, Minneapolis, USA (catalogue number DC3L10).

The Kolmogorov-Smirnov test was used to check the data distribution. All the quantitative variables were described in the form of medians and ranges. Comparisons between the groups were examined with the Mann–Whitney *U* test. Correlations between the variables were calculated using Spearman's rank correlation. *p* value less than 0.05 was considered to be statistically significant.

## 3. Results

**Figure 1:**
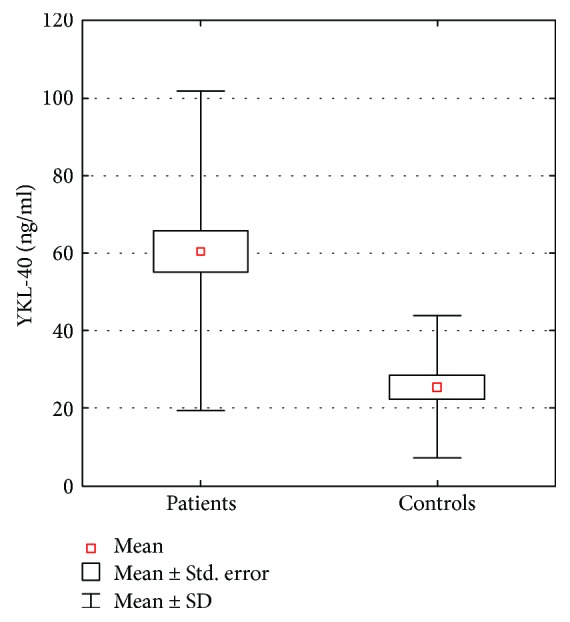
Comparison of serum YKL-40 concentration in patients with atopic dermatitis and in the control group.

**Figure 2:**
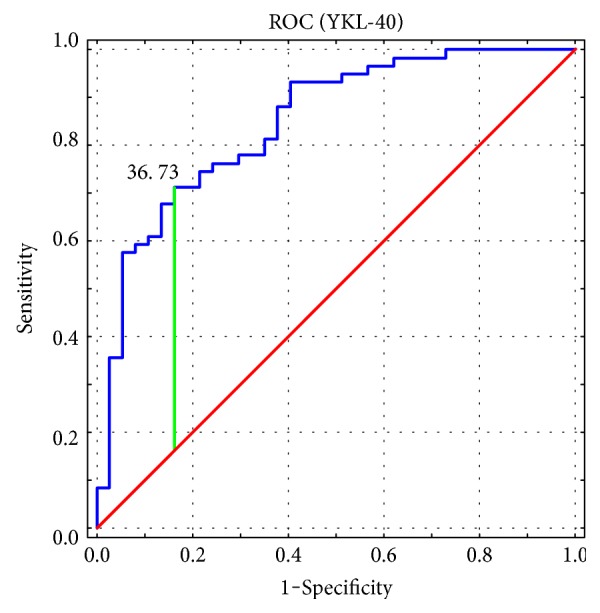
ROC analysis and the area under the curve (AUC) for YKL-40 serum level in patients with atopic dermatitis.

YKL-40 serum levels were significantly higher (*p* < 0.000001) in patients with AD, compared to those in the controls. The mean YKL-40 serum level in AD patients was 60.6 ± 41.2 ng/ml. Among the control subjects, the mean value of this parameter amounted to 25.5 ± 18.5 ng/ml. The difference in serum YKL-40 levels in AD patients and the control group is shown in Figure 1. ROC analysis presented the large area under the curve (AUC) (Figure 2). The optimal cut-off value for serum YKL-40 level was 36.73 with high negative (NPV) and positive predicting values (PPV) of 0.646 and 0.875, respectively.

Furthermore, we have found relationships between YKL-40 serum concentration, the severity of skin changes, and pruritus in patients with AD. There has been a significant positive correlation between YKL-40 level and both the SCORAD (*p* = 0.035) and objective SCORAD (*p* = 0.045) indexes. Moreover, serum YKL-40 level correlated significantly with the severity of pruritus measured by the VAS (*p* = 0.015). The presence of other atopic diseases in general did not influence YKL-40 serum level in atopic patients (*p* = 0.64). The additional separate analysis was performed for patients suffering from asthma and rhinitis, and none of these specific problems had an impact on YKL-40 serum concentrations. The detailed data are shown in Table 2.

The mean total IgE was quite high and amounted to 10989.4 ± 16763.2 IU/ml. Almost all patients had significantly elevated total IgE level. Only 7 patients (11.9%) had total IgE within the normal range (below 150 IU/ml). Eosinophil count exceeded the normal range only in 16 individuals (27%) with the mean value of 0.67 ± 1.31 × 10^3^/ul in the whole group of AD patients. CRP mean value was 6.18 ± 12.3 mg/ml and was elevated in 8 patients (13.5%). WBC amounted to the mean value of 8.56 ± 3.3 × 10^3^/ul and only 8 patients (13.5%) had this parameter above the normal range. Patients with elevated CRP and WBC had no signs of infection. All the laboratory results in the group of AD patients are shown in Table 3. There were no significant correlations between serum YKL-40 levels and laboratory parameters like age, gender, total IgE, eosinophilia, CRP, ESR, WBC, or neutrophil count (data not shown).

## 4. Discussion

This study has shown that YKL-40 may play a role in the pathology of AD. We have demonstrated not only that serum level of this protein is increased in patients suffering from AD but also that its concentration reflects the severity of skin changes. Moreover, it is also related to pruritus, which is the most important and essential subjective symptom in AD. The source of increased YKL-40 level is unknown, however it may be assumed that this protein is released by inflammatory cells activated in the course of the disease. The presented study is so far the first report focusing on the role of YKL-40 protein in skin atopy. However, the recent research has found the evidence of YKL-40 contribution to the mechanisms of type 2 allergic response and its association with atopy. It has been demonstrated, on the animal model, that YKL-40 is essential for the development of Th2 allergic inflammation. Genetically changed mice, carrying the YKL-40 null mutation, had a significant defect in antigen-provoked or IL-13-induced Th2 response; however, transgenic expression of YKL-40 in the epithelium was able to restore the process [13]. It was suggested that YKL-40, *i.a.*, contributes to inhibition of T-cells apoptosis and stimulates dendritic cells influx.

YKL-40 is encoded by the chitinase 3-like 1 gene (CHI3L1). Genetic studies on human CHI3L1 gene have revealed that some single nucleotide polymorphisms (SNPs) can be associated with a higher risk of atopy. The Korean study on children has demonstrated that some variations in the CHI3L1 promoter correlate with atopy [16]. The genetic polymorphisms of the CHI3L1 promoter are also responsible for the expression of YKL-40, which provides the evidence that this protein contributes to the atopic allergy [22]. Another study on a large cohort of adults with asthma has shown that some SNPs of the CHI3L1 gene are significantly associated with atopy [23]. All these studies give a new insight into the understanding of the role of YKL-40 in atopic allergy and validate the need for further investigation of this matter.

YKL-40 has been reported to be increased in patients with asthma and was also proved to be a biomarker of the severity of asthma, showing the correlation with some clinical parameters of the disease [22, 24–26]. Many authors concluded that YKL-40 is probably related to the inflammation and bronchial tissue remodeling, both being the essential components of asthma pathogenesis. However, some further studies on asthma have revealed particular associations of YKL-40 with atopic asthma. One of the reports has shown elevated serum YKL-40 in asthmatics in general, compared to healthy controls [27]. Furthermore, the same study has demonstrated significantly higher YKL-40 levels in the subgroup of atopic patients versus nonatopic asthmatics, showing a specific additional correlation with atopy. Another study was performed on the group of patients with atopic asthma and demonstrated increased YKL-40 levels in induced sputum; several hours after an allergen, bronchial provocation test inducing an allergic airway inflammation was performed [18]. These findings confirm a contribution of YKL-40 to pathogenesis of atopic asthma. However, the studies conducted so far have failed to find the correlation between YKL-40 and IgE or blood eosinophilia, which is in concordance with our results [25]. The contribution of YKL-40 to the atopic pathomechanisms has been also shown in allergic rhinitis [19]. The expression of YKL-40 was upregulated in epithelial cells in patients with persistent allergic rhinitis.

The study shows an elevated serum level of YKL-40 in patients with AD; however, the exact mechanisms underlying such an elevation are not clear. It remains uncertain whether the examined protein is related only to inflammation or is released also as a result of specific biochemical processes due to atopy. The study has also some limitations: the examined group of atopic patients has been rather small. Moreover, no relationships between serum YKL-40 and other biochemical markers of severity of atopic dermatitis and inflammation have been investigated.

## 5. Conclusions

The presented study is the first report showing an increased serum level of YKL-40 in patients with AD, observed regardless of the presence of other atopic diseases. The above described findings may constitute another evidence for the role of YKL-40 in atopic allergy but it still requires further investigations. Further research is also needed to establish whether YKL-40 can be used as a prognostic factor preceding the flares of AD.

## Figures and Tables

**Table 1:** Clinical characteristics of the examined group of patients with atopic dermatitis (*n* = 59).

Parameters	Mean ± SD (range)	Minimal value	Maximal value
Age (years)	32.8 ± 11.2	18	64
Duration of AD (years)	18.7 ± 13.5	0.5	59
SCORAD index	49.1 ± 16.3	12.8	85
Objective SCORAD	40.9 ± 14.5	8.8	76
Pruritus (VAS)	6.7 ± 2.2	0.8	10

**Table 2:** The analysis of YKL-40 serum level (ng/ml) in patients with AD and other concomitant atopic diseases.

	YKL-40 (mean ± SD)		Significance
	Presence	absence	
Atopic diseases in general (*n* = 33)	56.6 ± 33.9	65.7 ± 49.2	*p* = 0.64
Asthma (*n* = 13)	46.7 ± 21.7	64.5 ± 4.6	*p* = 0.24
Rhinitis (*n* = 27)	56.9 ± 35.6	63.7 ± 45.8	*p* = 0.64

**Table 3:** Laboratory findings in patients with atopic dermatitis (*n* = 59).

Parameters	Mean ± SD (range)	Minimal value	Maximal value
YKL-40, ng/ml	60.6 ± 41.2	15.05	213.22
IgE total, IU/ml	10,989.4 ± 16,763.2	5	88,500
CRP, mg/ml	6.18 ± 12.3	0.2	57.4
ESD, mm/h	11.9 ± 12.4	3	58
WBC, ×10^3^/ul	8.56 ± 3.3	4.02	18.2
Neutrophil count, ×10^3^/ul	5.2 ± 2.87	1.7	14.88
Eosinophilia, ×10^3^/ul	0.67 ± 1.31	0	9.1
